# Differential effects of radiation fractionation regimens on glioblastoma

**DOI:** 10.1186/s13014-022-01990-y

**Published:** 2022-01-25

**Authors:** Kelly J. McKelvey, Amanda L. Hudson, Heather Donaghy, Shihani P. Stoner, Helen R. Wheeler, Connie I. Diakos, Viive M. Howell

**Affiliations:** 1 Bill Walsh Translational Cancer Research Laboratory, Faculty of Medicine and Health, The University of Sydney, St Leonards, NSW 2065 Australia; 2 Northern Sydney Cancer Centre, Royal North Shore Hospital, St Leonards, NSW 2065 Australia; 3 Department of Medical Oncology, Royal North Shore Hospital, St Leonards, NSW 2065 Australia

**Keywords:** Inter-fractional, Radiation, Glioblastoma, Radiation biology

## Abstract

**Background:**

Radiotherapy (RT) is a mainstay of treatment for patients with glioblastoma (GB). Early clinical trials show that short course hypofractionation showed no survival benefit compared to conventional regimens with or without temozolomide chemotherapy (TMZ) but reduces the number of doses required. Concerns around delayed neurological deficits and reduced cognition from short course hypofractionated RT remain a concern. The aim of this study was to evaluate the effect of increased interfractional time using two different radiation fractionation regimens on GB.

**Methods:**

The radiobiological effect of increasing doses 0–20 Gy x-ray photon RT on Gl261 and CT2A GB cell lines was compared by colony forming assay, DNA damage by alkaline comet assay, oxidative stress, DNA damage, cell cycle, and caspase-3/7 by MUSE® flow cytometric analyses, and protein expression by western blot analyses. Conventional (20 Gy/10 fractions) and hypofractionated (20 Gy/4 fractions spaced 72 h apart) RT regimens with and without TMZ (200 mg/kg/day) were performed in syngeneic Gl261 and CT2A intracranial mouse models using the Small Animal Radiation Research Platform (Xstrahl Inc.).

**Results:**

X-ray photon radiation dose-dependently increased reactive oxygen species, DNA damage, autophagy, and caspase 3/7-mediated apoptotic cell death. While the conventional fractionated dose regimen of 20 Gy/10 f was effective at inducing cell death via the above mechanism, this was exceeded by a 20 Gy/4 f regimen which improved median survival and histopathology in Gl261-tumor bearing mice, and eradicated tumors in CT2A tumors with no additional toxicity.

**Conclusions:**

Spacing of hypofractionated RT doses 72 h apart showed increased median survival and tumor control via increased activation of RT-mediated cell death, with no observed increased in radiotoxicity. This supports further exploration of differential RT fractionated regimens in GB clinical trials to reduce delayed neurological radiotoxicity.

**Supplementary Information:**

The online version contains supplementary material available at 10.1186/s13014-022-01990-y.

## Background

High grade glioma remains a debilitating disease and devastating diagnosis for patients. Despite several clinical trials of checkpoint inhibitors, monoclonal and bispecific antibodies, vaccines and oncolytic viruses, and targeted therapies [1–3], external beam radiation therapy (EBRT) remains at the forefront of treatment for most patients in both the paediatric (i.e. diffuse midline gliomas) and adult high grade populations (i.e. Glioblastoma, GB). However, while EBRT is effective for irradiating the tumor bed post-surgery, managing symptoms, and temporarily disrupting tumor growth, most patients will eventually relapse and succumb to their disease.

In Australia, patients with primary high grade brain tumors, such as GB, are prescribed either (1) concurrent chemoradiotherapy consisting of 60 Gy in 30 fractions (60 Gy/30 f; daily 2 Gy) with adjuvant temozolomide (TMZ) chemotherapy, or (2) where patients are elderly, have significant co-morbidities, or cannot tolerate TMZ chemotherapy a 34 Gy/10 f or 25 Gy/5 f regimen (3.4 and 5 Gy daily respectively) [4]. Despite this, many patients relapse and the median survival for patients receiving the conventional 60 Gy/30 f European Organisation for Research and Treatment of Cancer (EORTC) protocol is ~ 16 months post-diagnosis [5]. Yet, equivalent or higher doses are prescribed for brain metastases from lung or breast cancer in both the treatment and palliative setting with some success [4]. Examples include stereotactic radiosurgery where 15–24 Gy is delivered in a single fraction; whole brain irradiation of 20 Gy/5 f (daily 4 Gy); or a hypofractionated RT regimen for brain metastases delivering 24–30 Gy in 3–5 fractions.

A recent meta-analysis by Trone and colleagues in first line GB revealed that hypofractionation lead to comparable survival outcomes—overall survival and progression free survival—with the benefit of a shortened duration of treatment [6]. Similar conclusions have been reported in the elderly GB [7] and paediatric diffuse midline glioma patient populations [8]. While hypofractionated RT regimens have been examined in the clinic, the mechanism of action from differential fractionated regimens are often not explored preclinically to elucidate wherein we may better reduce delayed neurological symptoms associated with radiotoxicity.

RT evokes inflammatory, coagulation and immune responses [9, 10] in a dose-dependent fashion. Yet, it also reduces the number of circulating lymphocyte populations [11, 12] which are integral to mounting an anti-tumor immune response. Lympho-depletion is dependent on the dose and exposure of circulating immune cells to RT as they pass through the RT field. Therein, a modified fractionated RT regimen may be more permissive of a strong, durable anti-tumor immune response. In addition, we explored a 72 h spacing between fractionated doses to enable DNA repair in healthy cells, and the accumulation of cancers into more radiosensitive phases of the cell cycle.

The mechanism of action of two fractionated EBRT regimens was examined in vitro and in vivo using the syngeneic allograft Gl261 and CT2A GB models. These models reflect several heterogenous aspects of the histopathology, molecular/mutational subtypes, radioresistance, chemoresistance and therapeutic responses of patient GB tumors, including differential mutational status of the TP53tumor suppressor gene (Gl261 P53^MUT^ and CT2A P53^WT^), in an immunocompetent model wherein the contribution of a complete immune system to fractionated RT is present [4, 13, 14].

## Methods

### Cells

Murine glioma Gl261 cells were donated by Géza Safrany (Frederic Joliot-Curie National Research Institute for Radiology and Radiohygiene, Hungary) and CT2A by Erica Wilson (Leeds Institute of Medical Research at St James's, University of Leeds, Leeds, UK). Glioma cells were cultured in Dulbecco’s Modified Eagle Medium supplemented with 10% v/v foetal bovine serum (FBS) in a humidified incubator with 95% v/v air and 5% v/v CO_2_ at 37ºC with 60% relative humidity. The cell lines were mycoplasma negative by MycoProbe® Mycoplasma Detection Kit (R&D Systems Inc, Minneapolis, MN, USA) and authenticated by Short Tandem Repeat profiling (Garvan Institute of Medical Research, Sydney, NSW, Australia) at the beginning and end of experimentation.

### Cell treatments

Antioxidant N-acetyl-L-cysteine (NAC; #A7250; Sigma-Aldrich, St. Louis, MO, USA) was used at 1 mM prepared in media. In vitro irradiation was performed using the Small Animal Radiation Research Platform (SARRP; Xstrahl Inc., Suwanee, GA, USA) at a dose rate of 2.97 Gy/min, as reported previously [14]. For immunoblot analyses cells were treated with 100 nM Bafilomycin A1 (#B1793; Sigma-Aldrich, St. Louis, MO, USA) for the last 4 h of the treatment period to block lysosomal degradation. Cells were detached with 0.5% v/v and trypsin/0.2% v/v ethylenediaminetetraacetic acid in phosphate buffered saline (PBS) and counted by MUSE® cell count and viability assay (Luminex Corp., Austin, TX, USA) according to the manufacturers’ instructions.

### Clonogenic/colony forming unit assay (CFU)

Cells were seeded at 4,000 cells per well in 6-well plates with 2 mL of DMEM/10% v/v FBS. After 10 days colonies were stained with crystal violet (0.5% w/v, 1:1 methanol: distilled water) and then imaged and quantitated using a vSpot® Spectrum ELISpot/FluoroSpot Reader System and software (Autoimmun Diagnostika Gmbh, Straßberg, Germany). From the clonogenic cell survival fractions the linear quadratic (LQ) equation using Prism version 8.4.0 for Windows (GraphPad, San Diego, California, United States) was applied to calculate the cell survival (S) α/β ratio and biologically effective dose as outlined below [15] and BED values summarised in Additional file 1: Table S1:

Clonogenic cell survival (S) from a single fraction of radiation was calculated by Eq. 1:

1 $$LQS\left( d \right) = exp\left( { - \alpha d - \beta d^{2} } \right)$$

where d = dose of one fraction.

Assuming sub-lethally damaged cells were repaired between fractions, the clonogenic survival (S) for a n-fractionated regimen was calculated by Eq. 2:

2 $$LQS\left( {n,D} \right) = \left[ {LQS\left( d \right)} \right]^{n} = \exp \left( { - \alpha D - \beta D^{\frac{2}{n}} } \right)$$

where n = number of fractions, d = dose of one fraction, and D = n*d.

Biologically Effective Dose (BED) used to evaluate the “physical dose" to achieve a biological effect was calculated by Eq. 3:

3 $$BED = D\left[ {1 + \frac{d}{\alpha /\beta }} \right]$$

where *n* = number of fractions, *d* = dose of one fraction, and *D* = *n* * *d*.

### Functional assays

Cells were seeded at 5 × 10^5^ cells per 25 cm^2^ cell culture flask, equilibrated overnight and then treated for 72 h. The Oxidative Stress (#MCH100111), Multi-color DNA Damage (#MCH200107), Cell Cycle (#MCH100106), Autophagy LC3 (#MCH200109), Annexin-V (#MCH100105), and Caspase 3/7 (#MCH100108) assays and protocols were carried out according to the manufacturers’ instructions. Cell events (1000–10,000 assay dependent) were acquired and analyzed using the MUSE® Cell Analyzer (Luminex Corp., Austin, TX, USA).

### Alkaline comet assay

Single and double nuclear DNA strand breaks were quantitated by alkaline comet assay as per the manufacturer’s instructions (#4250-050K; R&D Systems, Minneapolis, MN, USA). Briefly, cells were seeded at 5 × 10^5^ cells per 25 cm^2^ cell culture flask, equilibrated overnight and then treated for 72 h. Cells were prepared at 1 × 10^5^/ml in ice cold PBS, combined 1:10 with agarose and spread onto glass microscope slides. Cells were lysed and DNA unwound and electrophoresed in alkaline electrophoresis solution (300 mM NaOH, 1 mM EDTA, pH > 13) and stained with SYBR® Gold nucleic acid stain (Invitrogen, Carlsbad, CA, USA) in 10 mM Tris–HCl pH 7.5, 1 mM EDTA buffer. Micrographs of high-power fields (20 × magnification) were acquired using SPOT Advanced™ software (version 3.5.9 for Windows; Diagnostic Instruments Inc, Sterling Heights, MI, USA) on an IX70 inverted fluorescent microscope (Olympus, Tokyo, Japan) and comet parameters quantitated using CASP Comet Assay Software (version 1.2.3b1 for Windows; available at (http://casplab.com/); CaspLab, Warsaw, Poland) [16].

### Western blot

Cells were seeded at 5 × 10^5^ cells in T25 flasks with 5 ml media overnight. Cells were treated with 1 mM NAC and/or 0–20 Gy radiation for 72 h and then lysed using radioimmunoprecipitation assay lysis buffer, sonicated, and centrifuge at 14,000 rpm for 40 min to remove debris. Fifty µg protein in reducing sample buffer was loaded on 4–20% Mini-PROTEAN® TGX™ Precast protein gels and transferred to low autofluorescence PVDF membranes (Bio-Rad, Hercules, CA, USA). Primary antibodies were SQSTM1/p62 (1:1000; ab56416; Abcam, Cambridge, UK), LC3B (1:1000; 83506S; Cell Signalling Technology, Danvers, MA, USA), γH2A.X (1:1000; ab11174; Abcam, Cambridge, UK), caspase 3 (1:1000; ab214430; Abcam, Cambridge, UK) and β-actin (1:10,000; A1978; Sigma). Bands were detected with 1:10,000 goat-anti mouse DyLight™ 680 conjugated or donkey anti-rabbit DyLight™ 800 conjugated and images acquired and analyzed using the Odyssey CLx Near-Infrared Fluorescence Imaging System (LI-COR Biosciences, Lincoln, NE, USA). Fluorescent band intensity was data standardised to β-actin per sample and then expressed relative to 0 Gy. Intensity values are summarised in Additional file 2: Table S2.

### In vivo syngeneic intracranial GB models

The murine median survival study was reviewed, approved, and performed in accordance with the with the Northern Sydney Local Heath District Animal Ethics Committee guidelines, Royal North Shore Hospital, St Leonards, Australia (Approval #RESP/17/205) which enforces the New South Wales Animal Research Act 1985.

Eight-week-old male C57Bl/6 mice (20–26 g) were purchased from the Kearn’s Animal Facility, Australia. Mice were housed in Allentown individually ventilated cages (3–5 per cage) with cellulose bedding under Specific Pathogen Free conditions. Standard chow and water were available ad libitum and enrichment was provided in the form of autoclaved ice block sticks or straws. Rooms were temperature controlled (22 °C) and kept on 12-h light/dark cycle (7:00/19:00 h).

Mice were inoculated with 1 × 10^5^/2 μl murine glioma Gl261 or CT2A cells using a stereotactic frame, microinjection unit (David Kopf Instruments, Tujunga, CA, USA) and 5 μl syringe with custom 32G needle (Hamilton Company, Reno, NV, USA) into the right caudoputamen at mediolateral 2 mm, anteroposterior − 0.1 mm, dorsoventral 3.0 mm from Bregma under isoflurane anaesthesia (2% v/v per 1L oxygen i.h.) as previously described [17]. Mice were randomly assigned into one of 6 treatment groups (6 mice per group). Treatments were administered daily for 2 weeks (5/7 days) commencing at day 7 post-inoculation; TMZ (200 mg/kg/day intraperitoneally) was administered 1 h before C-RT (20 Gy/10 f) or H-RT (20 Gy/4 f). In vivo dose rate was 3.71 Gy/min using the SARRP (Xstrahl Inc., Suwanee, GA, USA), as reported previously [14, 17]. Mice were imaged by cone beam computed tomography at 1° per image (60 kV, 0.8 mA, 1.0 mm aluminium filter) and reconstructed using the 3D Slicer-based MuriPlan™ Treatment Planning Software version 3.0.0 supplied with the SARRP [18]. A 5 × 5 mm fixed radiation field was utilised for the planning target volume based on the inoculation coordinates and known growth characteristics of these tumors from previous T2-weighted MRI and histological studies [14, 19]. The 2 and 5 Gy doses were calculated using MuriPlan’s super-position convolution dose engine [20] and irradiation performed using an x-ray beam of 220 kV, 13.0 mA, and 0.16 mm copper filtration. A summary of the SARRP image-guided irradiation methdolofy is provided in Additional file 3: Fig S1.

Animal weight and well-being was assessed twice weekly, and mice were euthanised at humane endpoint or at long-term survival (100 days post-inoculation) by cardiac puncture under isoflurane anaesthesia (2% v/v per 1L oxygen i.h.) followed by cervical dislocation. No adverse events were encountered.

### Histopathology

Four micron section of paraffin embedded tissues were stained with Mayer’s hemotoxylin and eosin Y/erythrosin B staining, and Ki67 (0.084 μg/ml; 12,202; Cell Signalling Technologies, Danvers, MA, USA), γH2A.X (0.06 μg/ml; ab11174; Abcam, Cambridge, UK) and CD45 (0.019 μg/ml; 70,257; Cell Signalling Technologies, Danvers, MA, USA) and counterstained with Mayer’s hematoxylin nuclear stain as previously described [21]. Slides were imaged using an Aperio XT slide scanner, captured using Aperio ImageScope (Leica Biosystems, Wetzlar, Germany), and five high power images were assessed per sample (N = 6 brains per treatment group) using ImmunoRatio plugin (Seinajoki, Finland) for the open-source platform Fiji/ImageJ as previously described [17].

### Statistical analyses

Data is expressed as mean ± standard deviation (SD) or standard error of the mean (SEM), as appropriate. To determine the statistical difference between treatments two-way ANOVA test with Dunnett’s multiple comparisons test were performed for MUSE® functional assays; and one-way ANOVA test with Dunn’s multiple comparison test for CFU, Autophagy LC3, immunoblot fluorescent intensity, and immunohistochemical analyses. Tail DNA content for alkaline comet assay was compared by Kruskal–Wallis test with Dunn’s multiple comparisons test. Murine survival studies are expressed as Kaplan–Meier Curves and compared by Log-Rank (Mantel-Cox) test. All statistics were performed using Prism version 8.4.0 for Windows (GraphPad, San Diego, California, United States) with *p* value < 0.05 considered significant.

## Results

### Radiation impairs GB mitochondrial function

**Fig. 1:**
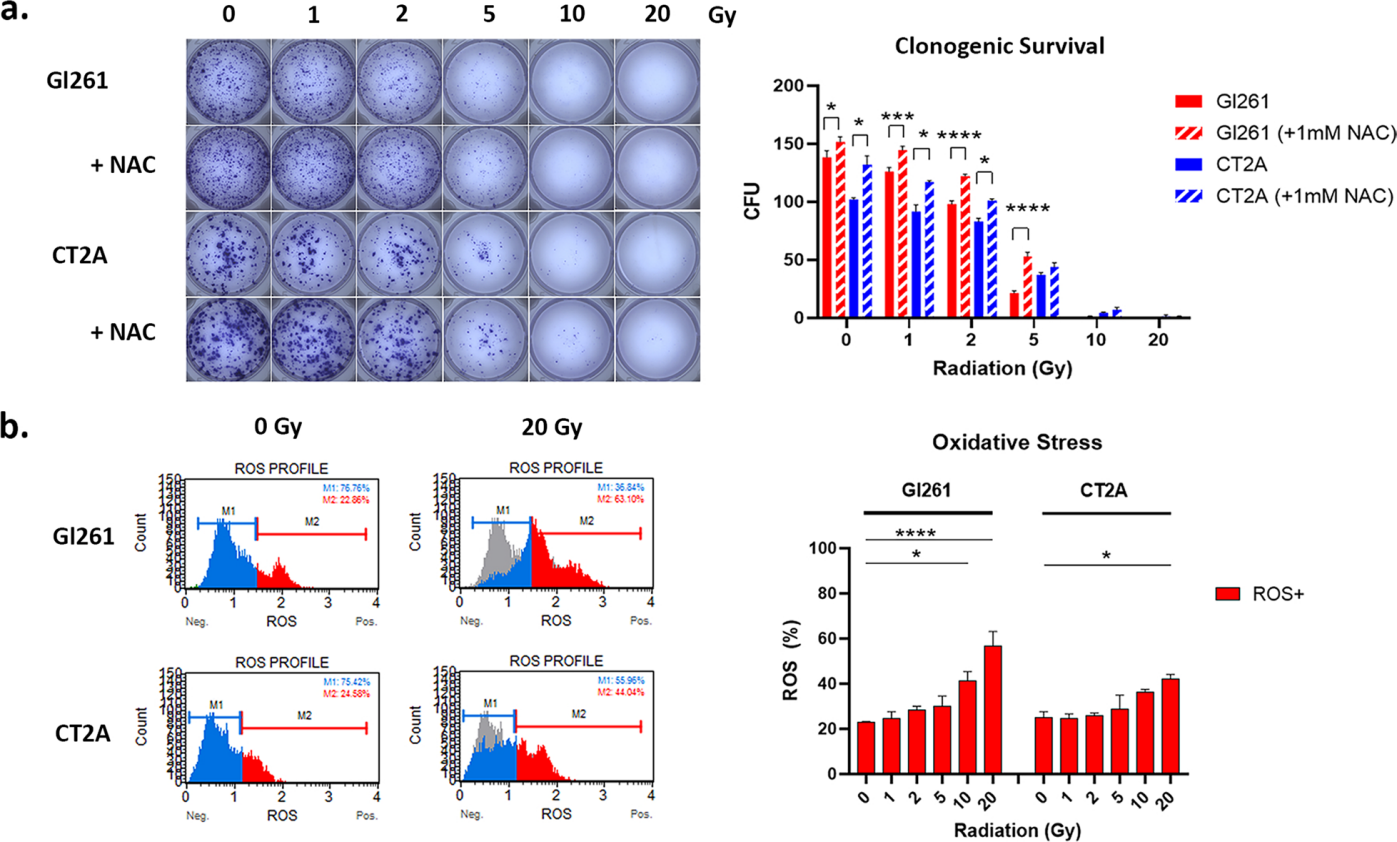
X-ray photon irradiation dose-dependently impairs GB mitochondrial function. **a** Micrographs of colony forming units (CFU; > 50 cells) 10-day post-RT with/out 1 mM NAC pre-treatment. Column graph shows mean CFU ± standard error of the mean (SEM) of triplicates from three independent experiments. **b** Histogram of ROS negative (blue) and positive (red) cells at 72 h post-irradiation. Grey overlay shows the profile at 0 Gy. Column graphs show mean percentage of ROS + cells (%) ± SEM of duplicates from three independent experiments. (**a** and **b**) *p < 0.05, **p < 0.01, ***p < 0.001, ****p < 0.0001 vs 0 Gy determined by two-way ANOVA with Dunnett’s multiple comparison test

To investigate whether a fractionated RT regimen with a 72 h inter-fractional space would be viable for GB we first examined the effect of single fractions of 0–20 Gy at 72 h. While 2 Gy was effective in reducing colony formation by 20–30% in both GB cell lines, increasing the dose to 5 Gy resulted in 60% to 85% inhibition of cell clonogenic survival (Fig. 1a). Pre-treatment with 1 mM of antioxidant NAC partially ameliorated this effect, demonstrating a role for ROS-mediated inhibition of GB clonogenic survival (Fig. 1a).

Unlike nuclear DNA, mtDNA is not shielded by histone proteins and supported by DNA repair processes, therefore we next examined the effect of increasing doses of radiation on mitochondrial function. Irradiation with 10–20 Gy significantly induced ROS at 72 h post-RT (Fig. 1b)., As ROS production is an initiating step of cell survival (e.g. autophagy) and cell death pathways (e.g. apoptosis) it suggests that doses higher than the standard 2 Gy fraction are required for cytotoxicity in GB cells.

### Radiation induced nuclear DNA damage and repair

**Fig. 2:**
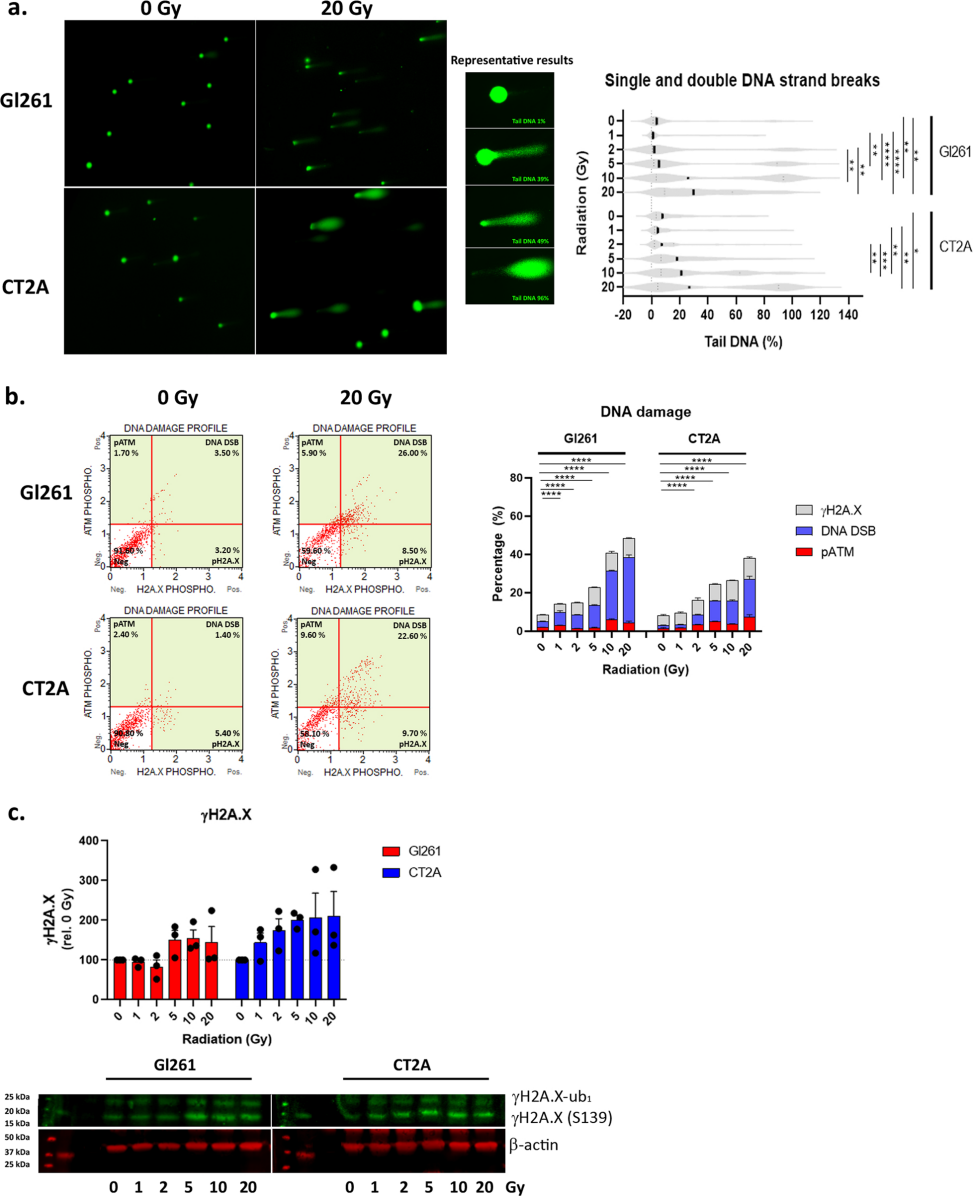
X-ray photon irradiation induced nuclear DNA damage and activated the DNA repair pathway. **a** Micrographs of single and double strand DNA breaks from alkaline comet assay at 72 h post-irradiation. Round cells indicate intact DNA, comet tails indicate DNA strand breaks. Violin plot shows median (solid line) length of comet tail (pixels) ± interquartile range (dotted line) of N = 50–73 comets per RT. *p < 0.05, **p < 0.01, ***p < 0.001, ****p < 0.0001 as determined by Kruskal–Wallis with Dunn’s multiple comparison test. **b** Dot plot of DNA repair indicate by phosphorylated ATM (pATM) and H2A.X (γ-H2A.X) at 72 h post-irradiation. Column graphs show mean percentage (%) ± SEM of duplicates from three independent experiments. *p < 0.05, **p < 0.01, ***p < 0.001, ****p < 0.0001 vs 0 Gy determined by two-way ANOVA with Dunnett’s multiple comparison test. **c** Immunoblot of γH2A.X (Ser139), mono-ubiquitinated γH2A.X (γH2A.X-ub_1_), and reference protein β-actin at 72 h post-irradiation. Band intensity was data standardised to β-actin per sample and then expressed relative to 0 Gy. Raw data is provided in Additional file 4: Fig. S2. Column graph shows mean fluorescent intensity relative to 0 Gy ± standard deviation (SD) of from three independent experiments

In addition to ROS production, irradiation induced dose-dependent increases in nuclear single and double DNA strand breaks were identified by the alkaline comet assay (Fig. 2a). Above 5 Gy the violin plots of box cell lines show the appearance of a second distribution of cells with increased DNA damage to the right of the primary population (Fig. 2a). This suggests that a subpopulation of cells acquired greater DNA damage with a higher dose. Median tail DNA content (%) showed a significant increase in DNA strand breaks with increasing radiation dose (Fig. 2a). In Gl261 cells there was no difference between 2 and 5 Gy (*p* > 0.99), while in CT2A cells this neared significance (*p* = 0.067). Following DNA damage, DNA repair enzymes are activated by phosphorylation. Activation of ataxia telangiectasia mutated (pATM) and histone 2A family member X (γH2A.X) was assessed by flow cytometry and indicated increasing DNA damage in a subset of GB cells with higher radiation doses (Fig. 2b); predominantly due to γH2A.X at the 72-h post-irradiation time point. To further validate γ-H2A.X up-regulation, we assessed protein expression by western blot analyses (Fig. 2c). This showed a stepped increase in γH2A.X protein expression at 5 Gy in Gl261 consistent with its IC50, while CT2A expression was dose dependent.

### DNA damage leads to cell cycle arrest, autophagy and apoptotic cell death

**Fig. 3:**
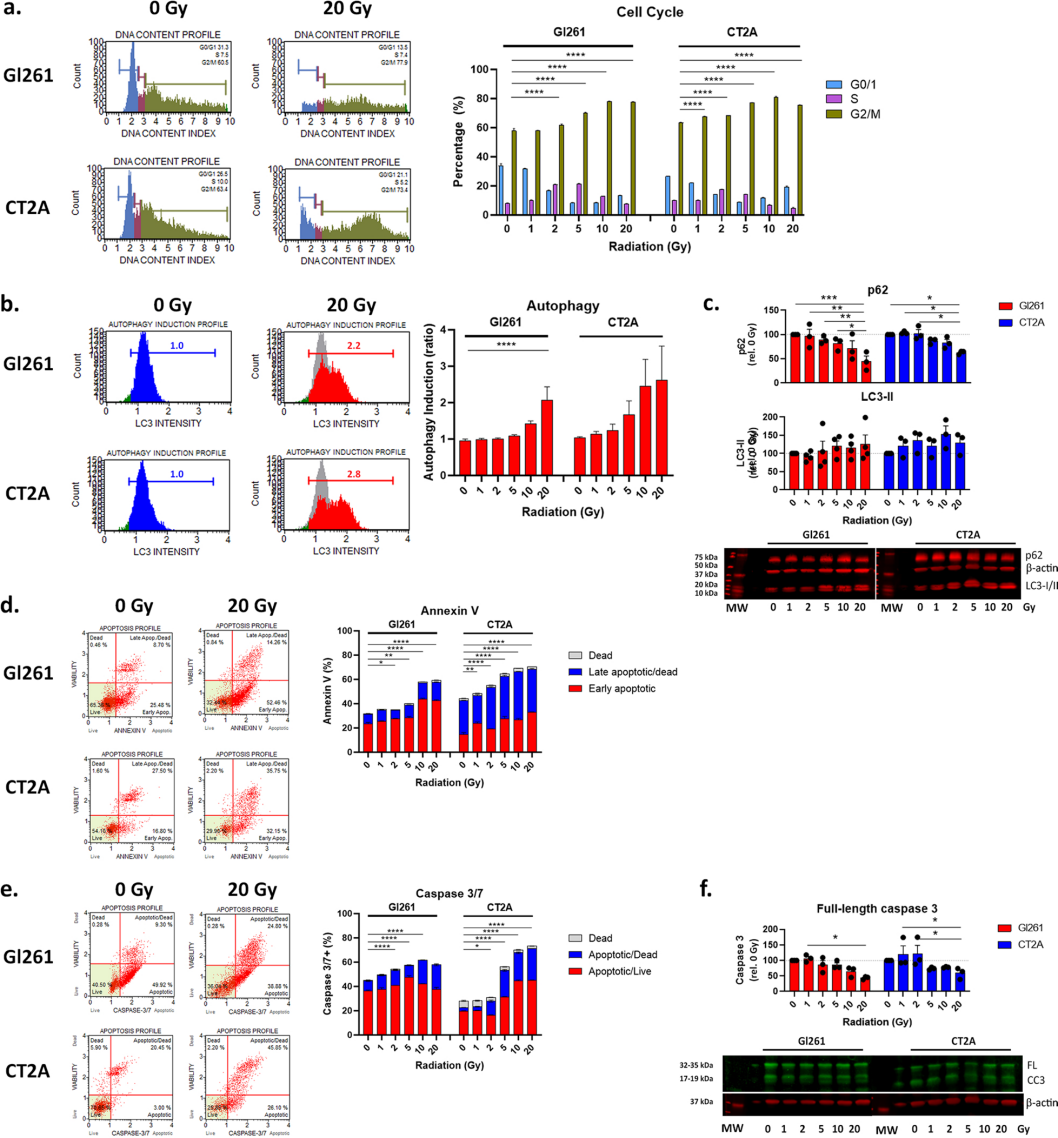
Radiation induced cell cycle arrest, autophagy and apoptotic cell death. **a** Histogram of DNA content in cells reflecting the G0/1, S, and G2/M phases of the cell cycle at 72 h post-irradiation. Stacked column graph shows mean percentage relative (%) ± SEM of triplicates from three independent experiments. **b** Histogram of autophagosome marker LC3B intensity in indicating the induction of autophagy following irradiation at 72 h post-irradiation. Grey overlay shows the profile at 0 Gy. Column graph shows mean ratio of autophagy induction relative to 0 Gy ± SEM of duplicates from three independent experiments. **c** Immunoblot of p62, LC3-I and II, and reference protein β-actin at 72 h post-irradiation. Band intensity was data standardised to β-actin per sample and then expressed relative to 0 Gy. Raw data is provided in Additional file 4: Fig. S2. Column graph shows mean fluorescent intensity relative to 0 Gy ± SD of from three independent experiments. **d** Dot plots of apoptotic marker annexin V and viability marker 7-AAD at 72 h post-irradiation. **e** Dot plots of apoptotic marker caspase 3/7 and viability marker 7-AAD at 72 h post-irradiation. Column and stacked column graphs show mean percentage (%) ± SEM of duplicates from three independent experiments. **f** Immunoblot of caspase 3 and reference protein β-actin at 72 h post-irradiation. Band intensity was data standardised to β-actin per sample and then expressed relative to 0 Gy. Raw data is provided in Additional file 4: Fig. S2. Column graph shows mean fluorescent intensity relative to 0 Gy ± SD of from three independent experiments. *p < 0.05, **p < 0.01, ***p < 0.001, ****p < 0.0001 vs 0 Gy determined by two-way ANOVA with Dunnett’s multiple comparison test

Damage to mtDNA and nuclear DNA leads to cell cycle arrest to enable DNA repair before cell division. We observed S-phase arrest in Gl261 and CT2A cells at 2 and 5 Gy (*p* < 0.0001; Fig. 3a) and G2/M cell cycle arrest at all doses > 1 Gy in both GB cell lines when compared to 0 Gy (*p* < 0.0001; Fig. 3a). Once entered in cell cycle arrest, cell may undergo DNA repair, autophagy to remove damaged organelles such as mitochondria, or apoptotic cell death. Increasing autophagy was indicated by increased LC3 intensity compared to 0 Gy for both GB cells lines (Fig. 3b). This was validated by decreasing protein expression of autophagy substrate p62 and increasing autophagosome marker LC3-II by western blot analyses (Fig. 3d). This indicates that GB cells utilise autophagy as a survival mechanism to avoid cell death when exposed to high doses of radiation.

To assess the induction of apoptotic cell death elicited by different radiation doses the early event of inversion of phosphatidylserine in the plasma membrane was assessed by annexin-V staining by flow cytometry (Fig. 3d). Akin to mitochondrial dysfunction and DNA damage, annexin-V-mediated apoptosis was induced at > 2 Gy in Gl261 and > 1 Gy in CT2A cells (Fig. 3d). Of note, phospholipid membrane inversion occurs in many cell death mechanisms—e.g. ferroptosis, necrosis, and apoptosis—therefore we examined activation of the caspase 3/7 pathway indicative of apoptotic cell death. Caspase 3/7-mediated cell death was significantly increased above 2 Gy in both GB cells by flow cytometry (Fig. 3e) and full-length caspase 3 protein expression significantly decreased in western blot analyses (Fig. 3f).

### Hypofractionated radiation significantly reduced GB cell survival

While 10 and 20 Gy are biologically effective in vitro they are unlikely to be suitable in fractionated regimens for brain cancer patients, due to radiation-induced cell injury to adjacent healthy brain parenchyma. Therein, we assessed a one-week fractionated regimen to compare the relative efficacy of 10 Gy/5 f vs 10 Gy/2 f. We first assessed the theoretical cell survival fractions and BED of the RT regimens (Additional file 1: Table S1) [15]. Not surprisingly, multi-fraction RT regimens reduced the cell survival fraction and increased the biologically effective doses compared to single-fraction doses (Additional file 1: Table S1). The data indicates that the 10 Gy/2 f regimen would deliver a ~ twofold higher BED than 10 Gy/5 f in both GB models. Therein we hypothesised that the 10 Gy/2 f would induce greater enhancement of the treatment pathway in vitro and greater reduction of tumor growth in vivo (Additional file 3: Fig. S2).

As radioresistance varies in different phases of the cell cycle with cells being most sensitive in the G2/M phase, and the single fraction data revealed that 5 Gy induced significant G2/M arrest at 72 h post-irradiation (Fig. 3a), we elected to irradiate the 10 Gy /2 f on day 1 and 4 (72 h between doses), while conventional 10 Gy/5 f was irradiated day 1–5 (24 h between doses), and perform analyses on day 8 i.e. 72 h after final irradiation.

**Fig. 4:**
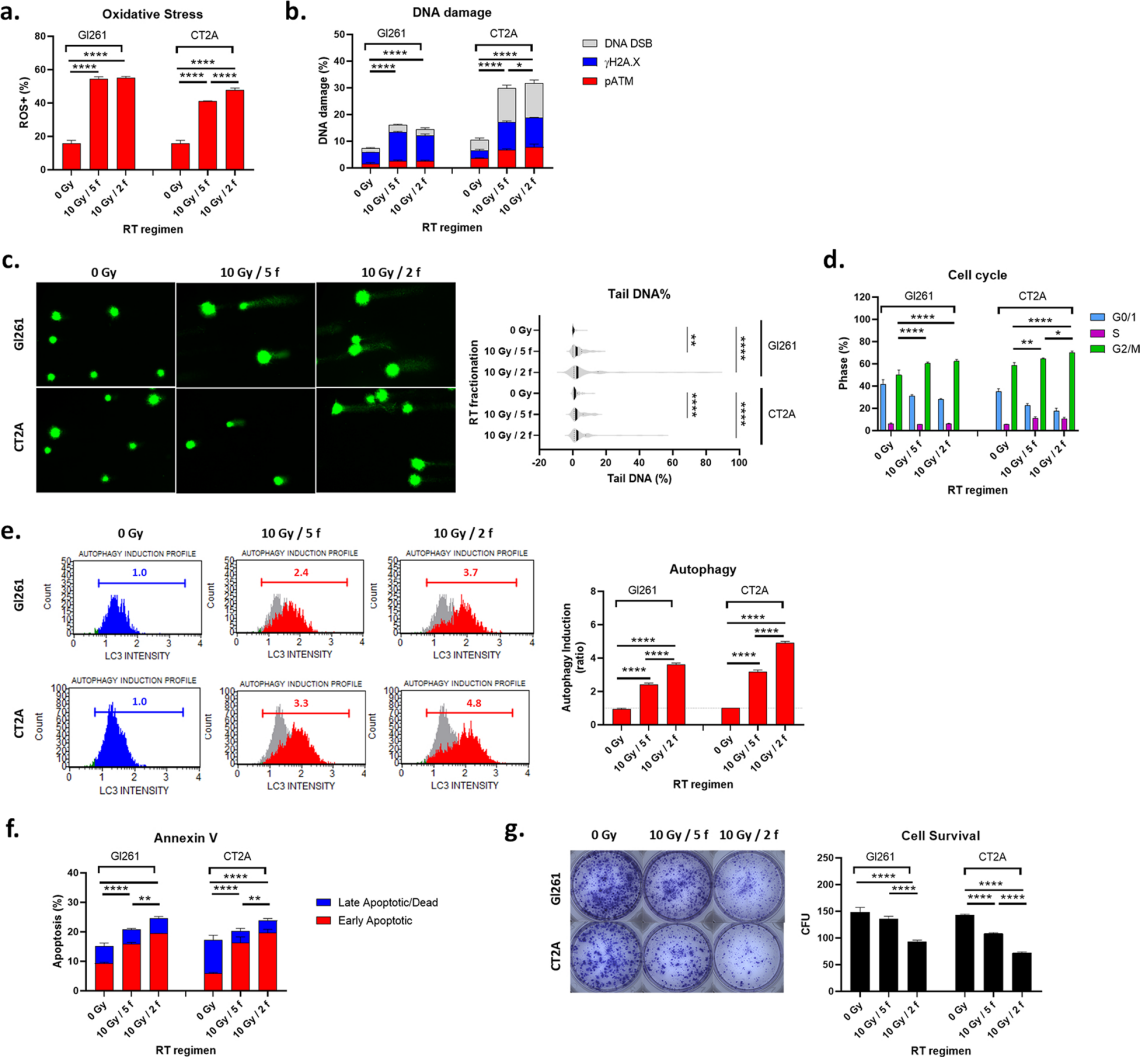
Hypofractionated RT is more effective than conventional fractionated RT. **a** Reactive oxygen species positive cells, **b** single and double DNA strand breaks, **c** phosphorylation of DNA repair proteins H2A.X and ATM, **d** cell cycle phase, **e** LC3-mediated autophagy, **f** annexin V-mediated apoptosis on day 8 (72 h after the last RT fraction), and **g** CFU on day 14 post-irradiation. (**a**, **b**, **d**–**g**) Column graphs show mean percentage (%) ± SEM of duplicates from three independent experiments. Grey overlay shows the profile at 0 Gy. *p < 0.05, **p < 0.01, ***p < 0.001, ****p < 0.0001 vs 0 Gy determined by one-way ANOVA with Tukey’s multiple comparison test. **d** Violin plot shows median (solid line) and interquartile range (dotted line) of tail DNA content as a percentage of total fluorescence for N = 46–55 comets per RT. **p < 0.01, ****p < 0.0001 vs 0 Gy determined by one-way Kruskal–Wallis test with Dunn’s multiple comparison test

Experimentally, both fractionated RT regimens increased oxidative stress (Fig. 4a), nuclear single and double strand breaks (Fig. 4b), nuclear DNA damage/repair enzymes (Fig. 4c), G2/M arrest (Fig. 4d), autophagy (Fig. 4e) and annexin V-mediated apoptotic cell death (Fig. 4f) when compared to untreated controls (0 Gy). Expectedly this was more pronounced in the more radiosensitive CT2A GB cell line. The hypofractionated RT regimen (10 Gy/2 f) significantly decreased cell survival in both cell lines when compared to conventional RT regimen (10 Gy/5 f; *p* < 0.0001; Fig. 4h). This suggests that increasing fractionated doses of RT from 2 to 5 Gy, is a potential treatment regimen for more radioresistant GBs.

### Hypofractionated radiation increased median survival in GB in vivo

**Fig. 5:**
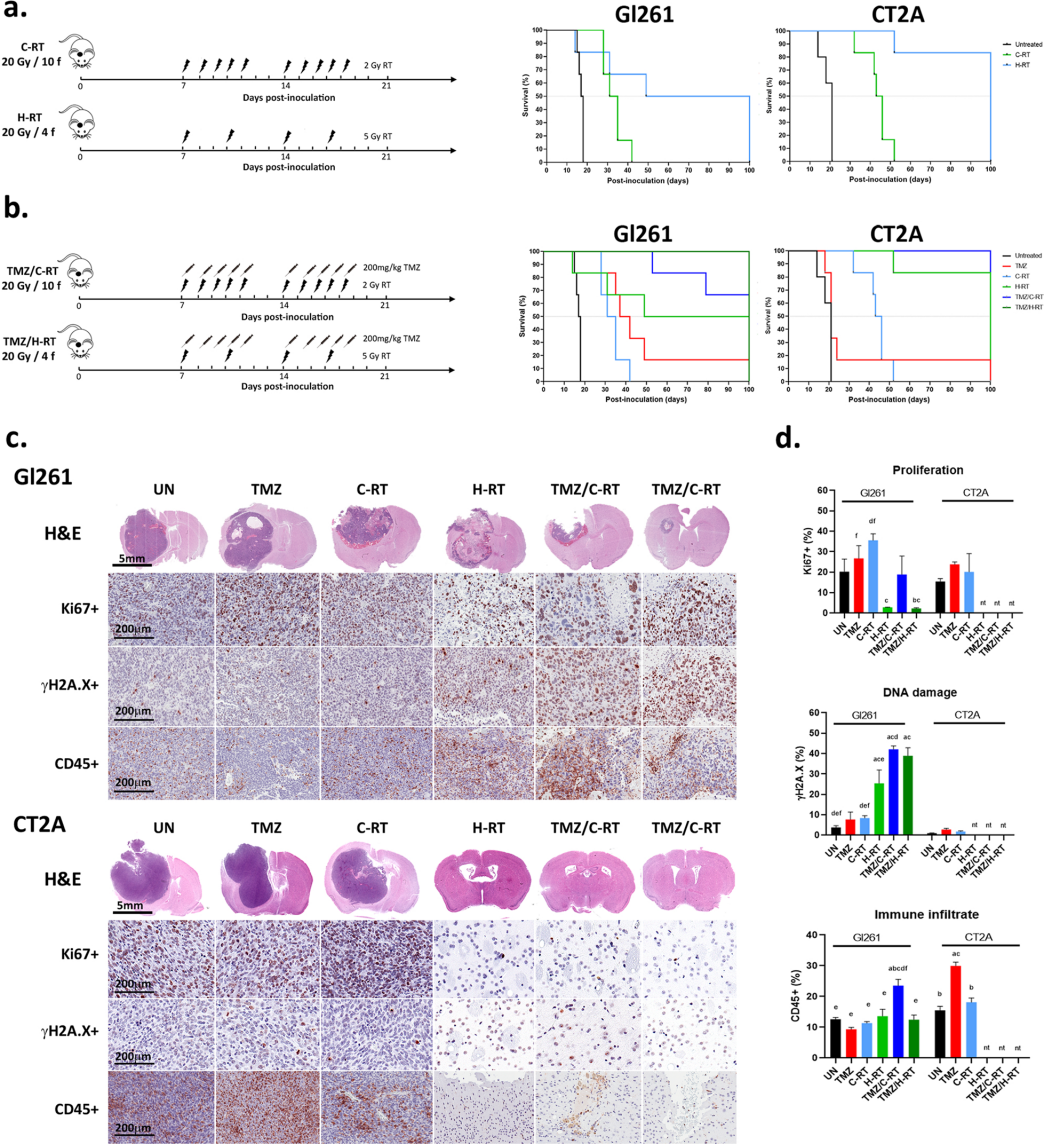
Hypofractionated RT regimen improves median survival and inhibits tumor proliferation in syngeneic GB models. **a** Schematic representation of the in vivo fractionated RT regimens with and without TMZ. **b** Kaplan–Meier plots show median survival of N = 6 mice per treatment group. **c** Micrographs of H&E and immunohistochemical staining for Ki67 + proliferation, γH2A.X DNA damage, and CD45 + immune cell infiltration. H&E scale bar = 5 mm, and histological micrographs = 200 µm. **d** Column graphs show the mean number of positive cells ± SEM per high power field. Five high power field were averaged per mouse. ^a^*p* < 0.05 vs untreated, ^b^*p* < 0.05 vs TMZ, ^c^*p* < 0.05 vs C-RT, ^d^*p* < 0.05 vs H-RT, ^e^*p* < 0.05 vs TMZ/C-RT, and ^f^*p* < 0.05 vs TMZ/H-RT determined by on-way ANOVA with Tukey’s multiple comparison test. nt, no discernible tumor

To determine whether the modified fractionated RT regimen could increase median survival of tumor-bearing animals, reduce tumor growth, and increase immunoreactivity (i.e. increase immune cell infiltration into tumors) without increasing radiotoxicity we utilised two immune competent GB models. Gl261 and CT2A cells were inoculated intracranial into syngeneic mice as previously described [17] and a two week treatment regimen initiated on day 7 post-inoculation to allow tumors to establish. Treatments comprised 20 Gy /4 f hypofractionated RT (H-RT) and 20 Gy/ 10 f conventional RT (C-RT) (Fig. 5a). H-RT significantly increased median survival compared to C-RT in both in vivo models (Gl261: 74.5 vs 33 days, *p* < 0.0001 and CT2A: 100 vs 44.5 days, *p* < 0.0001; Fig. 5a).

As RT is commonly provided concomitantly with TMZ chemotherapy for GB patients as part of the globally implemented EORTC protocol [5], both fractionated regimens were compared with daily 200 mg/kg TMZ chemotherapy (5/7 days per week; Fig. 5b) as previously reported [17]. CT2A tumor bearing animals were more ‘resistant’ to TMZ with no survival benefit compared to untreated tumors (Fig. 5b). However, when combined with either C-RT or H-RT, concomitant TMZ lead to 100% long term survival at 100 days post-inoculation in both models (Fig. 5b).

To compare the relative efficacy of the two fractionated RT regimens in more detail we performed histopathological analyses of tumor morphology, tumur proliferation and DNA damage (Fig. 3c). As noted in our previous study, Gl261 tumors developed focal necrosis and areas of hemorrhage following C-RT [17]. In Gl261 tumors this was more pronounced, with an enlarged tumor core of necrosis, but live peripheral tumor cells leading to mass effect within the brain and the death of 3 of the 6 mice in this treatment group. This is consistent with Gl261 tumors being more radioresistant than CT2A [17]. Concomitant TMZ and fractionated RT regimens showed reduced tumors in the mice bearing Gl261 tumors at 100 days compared to untreated controls, however, all CT2A inoculated mice in these treatment groups showed only signs of the needle tract and no discernible tumors.

Immunohistochemical analyses confirm a proliferating edge in H-RT treated tumors though overall there was a marked reduction in Ki67 + proliferation and increased DNA damage indicated by phosphorylation of DNA repair protein H2A.X in Gl261 tumors (Fig. 5c, d). Despite TMZ-resistance (inferred from the survival curve), CT2A tumors revealed a significant increase CD45^+^ immune infiltration of the tumors indicating greater immunoreactivity (Fig. 5d). In animals where the tumors were not discernible following the two fractionated RT regimens, there was no pathohistological evidence of RT-induced necrosis in the ‘normal brain parenchyma’ at 80 days post-irradiation (100 days post-inoculation) in the hematoxylin and eosin sections (Fig. 5c). Of note, CD45 + immune infiltrates were discernible along the residual needle track; however these may equally be attributable a persistent wound-healing response (Fig. 5c).

## Discussion

RT is a critical component of the EORTC protocol [5] that remains the most effective treatment for GB ~ 30 years after being assessed in clinical trials. In the EORTC protocol, 60 Gy is delivered in 30 fractions of 2 Gy per day over 6 weeks. We previously reported that RT of non-cancerous tissue can alter cell proliferation, blood vasculature and immunity in the sub-acute period (28 days) after conventional fractionated RT [21]. The current study assessed the biological effect of higher RT doses with fewer fractions (10 Gy/2 f per week) could reduce tumor growth, induce greater anti-tumor immunoreactivity, and minimise delayed radionecrosis compared to a conventional fractionated regimen (10 Gy/5 f per week).

**Fig. 6:**
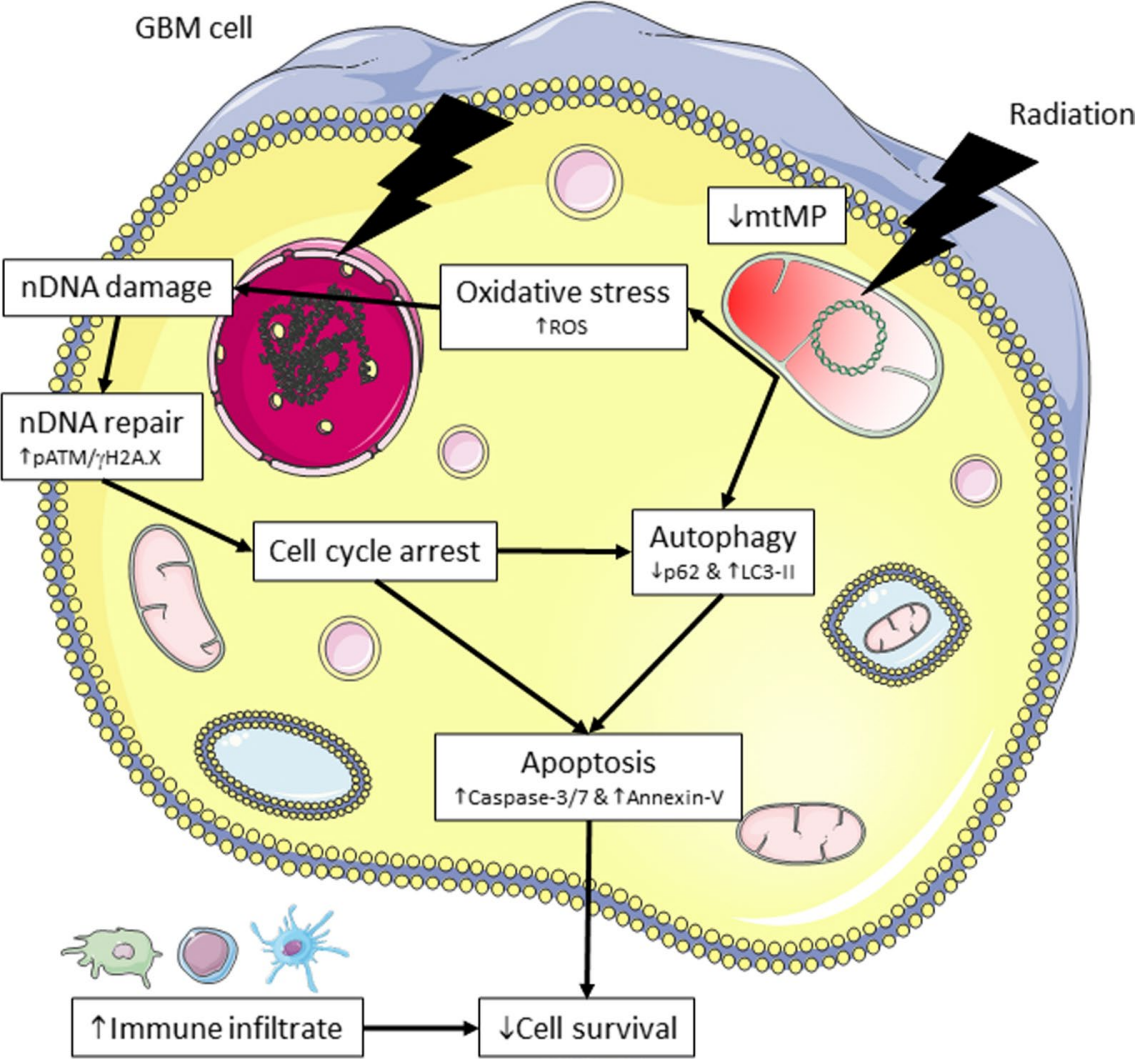
Mechanism of action of fractionated RT. Conventional and hypofractionated regimens further increase the oxidative stress, mitochondrial dysfunction, autophagy and apoptotic-mediated cell death to inhibit GB cell survival. mtDNA, mitochondrial DNA; nDNA, nuclear DNA. Figure was prepared using Servier Medical Art (https://smart.servier.com/), accessed on 3rd June 2021

Using in vitro and in vivo methods we demonstrate that whilst both fractionated regimen can significantly reduce tumor growth via the upregulation of oxidative stress, DNA damage, and induction of apoptotic cell death this was enhanced in the hypofractionated RT regimen in vitro (Fig. 6). In vivo the hypofractionated RT reduced tumor proliferation (Ki67) and tumor volume in Gl261 tumor-bearing mice and eliminated CT2A tumors.

Clinically, up to ~ 40% of GB patients progress after completing a conventional fractionated EBRT regimens [4], with 80% occurring locally with the 95% isodose RT field [22]. The use of protracted radiation schedules is believed to contribute to a lack of tumor control due to the repopulation of more radio-resistant tumor cells (e.g. glioma stem cells) [23]. Using our modified fractionated RT regimen we would propose to maintain the 6 week length of the GB patient RT course, however alter the 2 Gy daily fractions to 5 Gy bi-weekly fractions separated by 3 days (72 h). This could be alternatively modelled to achieve equivocal BED to the conventional fractioned RT regimen. By increasing the RT fraction dose from 2 to 5 Gy we achieved greater oxidative stress, cell kill, and push additional GB cells into the more radiosensitive G2/M phase of the cell cycle—at which point the next RT dose fraction is delivered (i.e. 72 h). A meta-analysis of hypofractionated RT with temozolomide in newly diagnosed GB patients found that hypofractionated RT increased overall and progression-free survival compared to conventional RT, with high-BED schedules improving 6- and 12-month progression-free survival over low-BED regimens [24]. Even moderate increases in fractional doses from 2 Gy to 2.2 or 2.4 Gy report good overall and progression-free survival with intensity-modulated radiotherapy regimens [25]*.*One of the main concerns for utilising higher RT doses in fractionated regimens is increased radionecrosis and acute or long-term neurocognitive decline and neuroendocrine dysfunction. Fractionated RT regimens have demonstrated less radionecrosis compared to single-fraction radiosurgery [26], yet there is a limited information on radionecrosis from conventionally fractionated vs differential fractionated RT regimens. Similar clinical regimens to our preclinical schedule have reported no significant radiotoxicity—30 Gy/6 f over 2 weeks [24, 25, 27–29]—and we did not observe histological changes in irradiated brain tissue of ‘tumor-free’ animals at 80 days post-irradiation. Continuation of our fractionated RT regimen for 6 weeks is yet to be explored as the aggressive nature of syngeneic GB tumors prohibits a treatment course of this duration. Patient-derived xenografts with longer survival are plausible though the absence of T-cells for the radioresponse and immunoreactivity in utilising immunocompromised or humanised mice cannot be yet equated to complete murine or human immune systems—therein interpretation of the experimental results in these models, at present, remains a question [29].

For gliomas, the survival of stem cell populations with capacity for self-renewal and tumor relapse remains a key concern for single and fractionated RT regimens. Treatment-induced autophagy in glioma stem cells is thought to be a contributing factor to tumor relapse [30]. We observed increasing levels of autophagy in the GB population following increasing doses of single fraction RT. Equally, both the conventional and modified fractionated RT regimens significantly upregulated autophagy. Autophagy serves as a critical survival mechanism by cells remove damaged organelles including mitochondria (termed mitophagy), the primary powerhouse of the cell. This can enable GB cells to overcome mtDNA damage, wherein mitochondria do not possess the ability to repair DNA, as for nuclear DNA. This may suggest that the addition of autophagy inhibitors to fractionated regimens is a viable therapeutic strategy warranting further investigation. Akin, to proposals for the combination of fractionated RT regimens with DNA repair inhibitors.

This study presents preclinical data that differential fractionated RT regimens to increase the interfractional time, rather than reducing the treatment course or interfractional time (i.e. bi-daily dosing) may be an avenue for further exploration to maintain tumor control, but more importantly reduce delayed radiotoxicity.

## Conclusions

Increasing the interfractional time between doses may enable higher RT fractional doses to utilised. This may increase tumor control and immunoreactivity, while avoiding long-term radiotoxicity and neurocognitive decline.

## Supplementary Information

**Additional file 1. Table S1**: Single and multi-fraction cell survival (S) and biological effective dose (BED).

**Additional file 2. Table S2**: Fluorescent band intensity for western blots.

**Additional file 3. Fig. S1**: Schematic of the SARRP image-guided irradiation methodology. Animals are imaged by cone beam computed tomography (CBCT) and then Hounsfield unit-based tissue segmentation. Dose planning for brain tumors is performed based on the inoculation coordinates and known tumor growth characteristics, and tumors irradiated with 2 or 5 Gy per dose fraction. Purple lines denote the radiation field from the target isocentre (IsoC). For high throughput studies, dose evaluations are performed retrospectively based on tumor location, dose planning, and treatment delivered.

**Additional file 4. Fig. S2**: Single cell stains for DNA damage and Annexin-V MUSE assays. Cells are gated for cells size to exclude debris (red rectangle; top) then cell populations gated against fluorophores (quadrants; bottom). (a) Dot plots of cell size and DNA damage - p-H2A.X-PE/Cy5 and pATM-PE – 1 hr post 20 Gy irradiation of Gl261 cells. (b) Dot plots of cell size and viability (7-AAD) against phosphatidylserine membrane translocation (Annexin-V-PE) in untreated Gl261 cells after 72 hrs. Note, the 7-AAD and Annexin-V dyes in the MUSE kit are provided as a combination dye, so single stain of annexin-V-PE is not available (N/A). The 7-AAD dye from another MUSE kit was used to provide a 7-AAD single stain.

## Data Availability

All data generated or analysed during this study are included in this published article.

## Abbreviations

BED: Biological effective dose
C-RT: Conventional RT regimen
DNA: Deoxyribonucleic acid
EBRT: External beam radiation therapy
EORTC: European Organisation for Research and Treatment of Cancer
F: Fraction
GB: Glioblastoma
γH2AX: Gamma histone 2A family member X
Gy: Gray
H-RT: Hypofractionated RT regimen
LQ: Linear quadratic
pATM: Phosphorylated ataxia telangiectasia mutated
RT: Radiation therapy
S: Survival
SD: Standard deviation
SEM: Standard error of the mean
TMZ: Temozolomide
