# Supplementary material for: Differential effects of radiation fractionation regimens on glioblastoma
Source: Radiat Oncol. 2022 Jan 25;17:17. doi: 10.1186/s13014-022-01990-y (PMC8788072; doi:10.1186/s13014-022-01990-y)
Supplement: Supplementary file 1 — Additional file 1. Table S1: Single and multi-fraction cell survival (S) and biological effective dose (BED). [file 13014_2022_1990_MOESM1_ESM.docx]

**Supplementary Table 1. Single and multi-fraction cell survival (S) and biological effective dose (BED)**

|  | **Cell Survival Fraction (S)** | | | |
| --- | --- | --- | --- | --- |
|  | **2 Gy** | **5 Gy** | **10 Gy / 5 f** | **10 Gy / 2 f** |
| **Gl261** | 0.73 | 0.54 | 0.48 | 0.38 |
| **CT2A** | 0.23 | 0.04 | 0.34 | 0.16 |
|  | **Biologically Effective Dose (BED)** | | | |
|  | **2 Gy** | **5 Gy** | **10 Gy / 5 f** | **10 Gy / 2 f** |
| **Gl261** | 4.94 | 8.95 | 24.72 | 44.77 |
| **CT2A** | 23.40 | 48.46 | 46.80 | 96.92 |

*Gy, gray; f, fraction.*
