# Supplementary material for: Differential effects of radiation fractionation regimens on glioblastoma
Source: Radiat Oncol. 2022 Jan 25;17:17. doi: 10.1186/s13014-022-01990-y (PMC8788072; doi:10.1186/s13014-022-01990-y)
Supplement: Supplementary file 3 — Additional file 3. Fig. S1: Schematic of the SARRP image-guided irradiation methodology. Animals are imaged by cone beam computed tomography (CBCT) and then Hounsfield unit-based tissue segmentation. Dose planning for brain tumors is performed based on the inoculation coordinates and known tumor growth characteristics, and tumors irradiated with 2 or 5 Gy per dose fraction. Purple lines denote the radiation field from the target isocentre (IsoC). For high throughput studies, dose evaluations are performed retrospectively based on tumor location, dose planning, and treatment delivered. [file 13014_2022_1990_MOESM3_ESM.pdf]

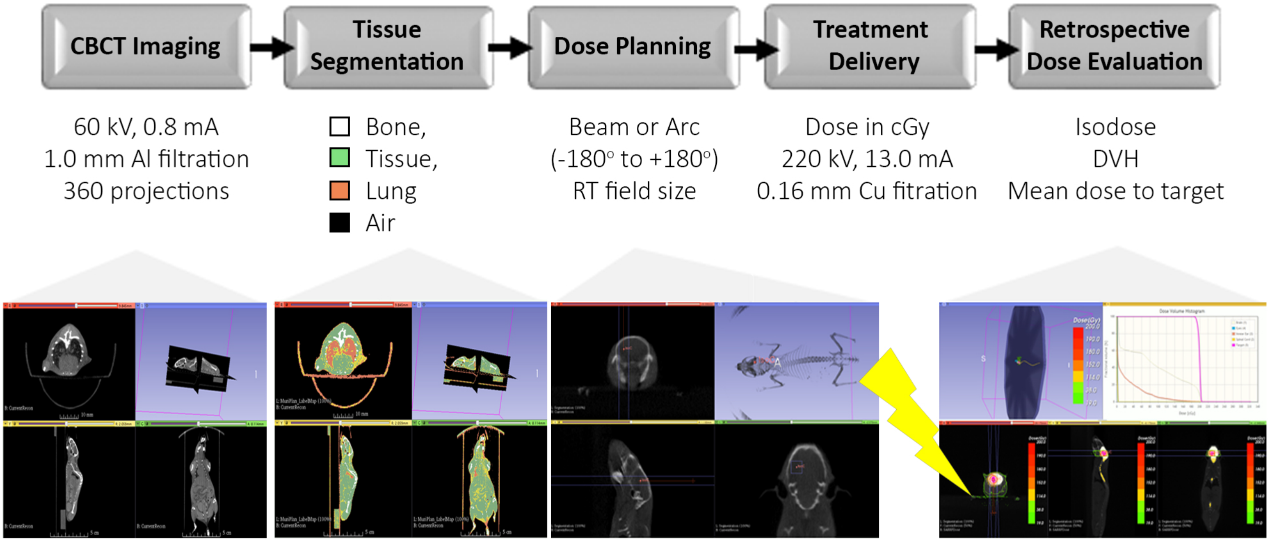
CBCT Imaging
Tissue Segmentation
Dose Planning
Treatment Delivery
Retrospective Dose Evaluation
60 kV, 0.8 mA
1.0 mm Al filtration
360 projections
Bone,
Tissue,
Lung
Air
Beam or Arc
(-180° to +180°)
RT field size
Dose in cGy
220 kV, 13.0 mA
0.16 mm Cu fitration
Isodose
DVH
Mean dose to target
