# Supplementary material for: Differential effects of radiation fractionation regimens on glioblastoma
Source: Radiat Oncol. 2022 Jan 25;17:17. doi: 10.1186/s13014-022-01990-y (PMC8788072; doi:10.1186/s13014-022-01990-y)
Supplement: Supplementary file 4 — Additional file 4. Fig. S2: Single cell stains for DNA damage and Annexin-V MUSE assays. Cells are gated for cells size to exclude debris (red rectangle; top) then cell populations gated against fluorophores (quadrants; bottom). (a) Dot plots of cell size and DNA damage - p-H2A.X-PE/Cy5 and pATM-PE – 1 hr post 20 Gy irradiation of Gl261 cells. (b) Dot plots of cell size and viability (7-AAD) against phosphatidylserine membrane translocation (Annexin-V-PE) in untreated Gl261 cells after 72 hrs. Note, the 7-AAD and Annexin-V dyes in the MUSE kit are provided as a combination dye, so single stain of annexin-V-PE is not available (N/A). The 7-AAD dye from another MUSE kit was used to provide a 7-AAD single stain. [file 13014_2022_1990_MOESM4_ESM.pdf]

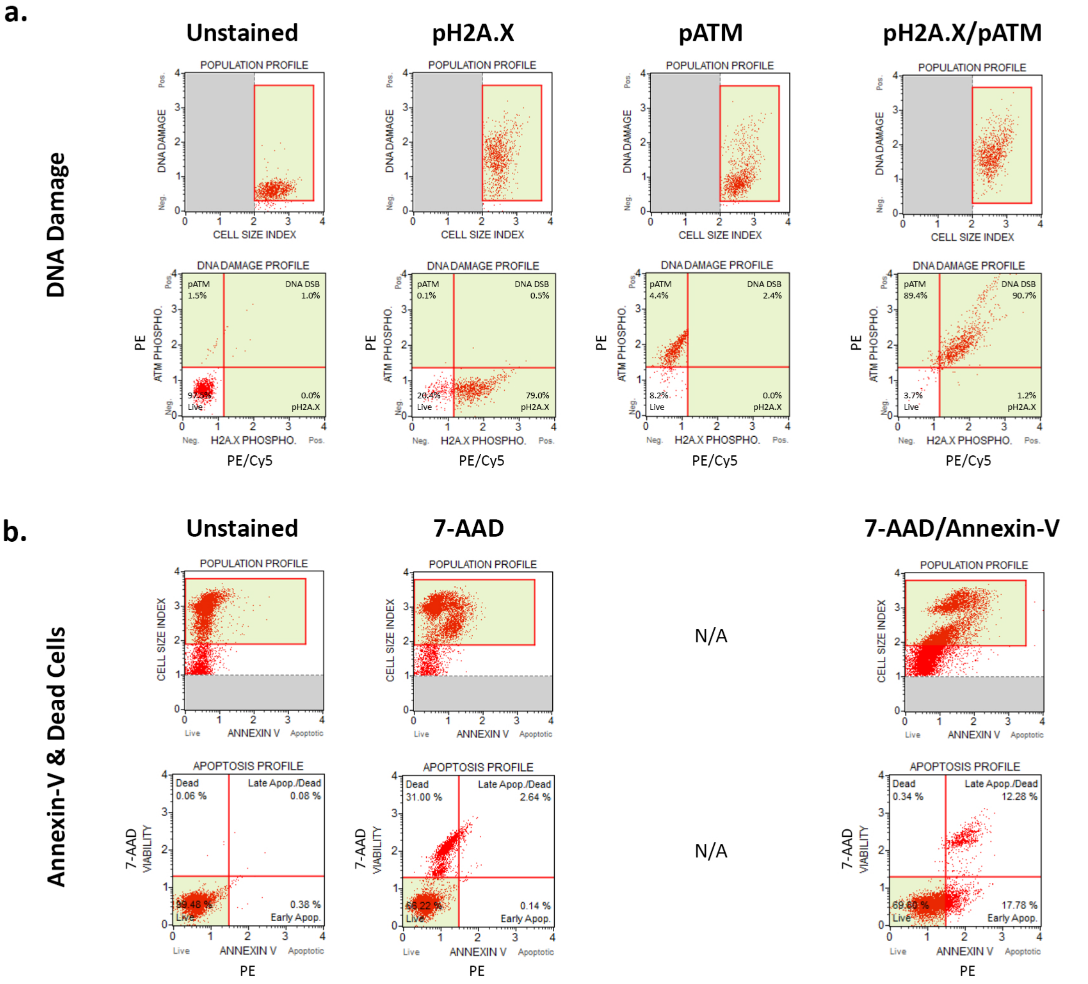

a.
DNA Damage
Unstained
pH2A.X
pATM
pH2A.X/pATM
POPULATION PROFILE
DNA DAMAGE
CELL SIZE INDEX
DNA DAMAGE PROFILE
ATM PHOSPHO.
H2A.X PHOSPHO.
PE
PE/Cy5
pATM 1.5%
DNA DSB 1.0%
97.5% Live
0.0% pH2A.X
pATM 0.1%
DNA DSB 0.5%
20.4% Live
79.0% pH2A.X
pATM 4.4%
DNA DSB 2.4%
8.2% Live
0.0% pH2A.X
pATM 89.4%
DNA DSB 90.7%
3.7% Live
1.2% pH2A.X
b.
Annexin-V & Dead Cells
Unstained
7-AAD
7-AAD/Annexin-V
POPULATION PROFILE
CELL SIZE INDEX
ANNEXIN V
Live
Apoptotic
N/A
APOPTOSIS PROFILE
7-AAD
VIABILITY
Dead 0.06 %
Late Apop./Dead 0.08 %
99.48 % Live
0.38 % Early Apop.
Dead 31.00 %
Late Apop./Dead 2.64 %
66.22 % Live
0.14 % Early Apop.
Dead 0.34 %
Late Apop./Dead 12.28 %
69.60 % Live
17.78 % Early Apop.
PE
